# Expression of CD4+ and CD8+ Tumor-Infiltrating Lymphocytes in Oral Squamous Cell Carcinoma and Their Relationship With Clinicopathological Parameters: A Cross-Sectional Study

**DOI:** 10.7759/cureus.58748

**Published:** 2024-04-22

**Authors:** S Marytresa Jeyapriya, A Mathan Mohan, M Sathish Kumar, R Madhavan Nirmal

**Affiliations:** 1 Oral Pathology and Microbiology, Karpaga Vinayaga Institute of Dental Sciences, Chengalpet, IND; 2 Oral and Maxillofacial Surgery, Karpaga Vinayaga Institute of Dental Sciences, Chengalpet, IND; 3 Oral and Maxillofacial Pathology, Rajah Muthiah Dental College and Hospital, Chidambaram, IND

**Keywords:** tumor-infiltrating lymphocytes, immune response, t-cell-mediated immune response, oral cancer, cancer, metastasis, tumor microenvironment

## Abstract

Background

Oral squamous cell carcinoma (OSCC) is the most common malignant neoplasm of the oral cavity. The tumor microenvironment (TME) is a dynamic ecosystem composed of components contributed by both the tumor and the host. The immune cells of TME, mainly CD4+ and CD8+ tumor-infiltrating lymphocytes (TILs), suppress the proliferation of cancer cells and play a crucial role in the progression of OSCC. The present study aims to analyze the immunohistochemical expression of CD4+ and CD8+ TILs in OSCC and to compare and correlate them with clinicopathological parameters.

Methodology

A total of 75 formalin-fixed paraffin-embedded samples of cases diagnosed with primary OSCC were immunostained with CD4+ and CD8+ antibodies and their expression was compared with the clinicopathological parameters.

Results

There was a significant positive correlation between CD4+ and CD8+ expression (r = 0.655, p = 0.001). Both CD4+ (r = -2.37, p = 0.041) and CD8+ (r = -0.348, p = 0.002) expressions negatively correlated with the TNM stage (r = -2.37, p = 0.041) of OSCC. CD8+ expression positively correlated with histopathological grade (r = 0.288, p = 0.012).

Conclusions

The study findings suggest that CD4+ cells are essential to maintain and sustain CD8+ TIL-mediated anti-tumor response. CD4+ and CD8+ TILs are key players in cell-mediated adaptive immunity and prevent tumor progression and metastasis. Strikingly, the higher grade of tumors despite heavy CD8+ infiltration may possibly be due to cancer immunoediting.

## Introduction

Oral squamous cell carcinoma (OSCC) is the eighth most common malignant neoplasm of the oral cavity worldwide. In India, it accounts for 30% of all cancer cases [1,2]. Despite over 50% of cases being diagnosed early, OSCC exhibits a high recurrence rate and low five-year survival, which could be attributed to local or distant metastasis [2].

The tumor microenvironment (TME) is a dynamic ecosystem created by cancer cells composed of components contributed by both the tumor and the host. This ecosystem acts as a storehouse of factors, including chemokines, growth factors, and cytokines. The dynamic niche of this ecosystem is required for tumor transformation. It is composed of acellular components, such as extracellular matrix and basement membrane, and cellular components, which include immune cell populations and non-immune cell populations. The immune cells include tumor-associated macrophages, tumor-associated neutrophils, dendritic cells, regulatory T cells (Tregs), natural killer cells, and tumor-infiltrating lymphocytes (TILs) [3]. The immune cells of the TME, mainly CD4+ and CD8+ TILs, suppress the proliferation of cancer cells and play a crucial role in cancer progression. TME can serve as an effective immunomodulatory target for anti-cancer therapies [4,5].

There is a gap in the literature on the role of TME, especially the correlation between CD4+ and CD8+ TILs with clinicopathological parameters in OSCC. Studying the expression of these cells and their correlation with clinicopathological parameters in OSCC can provide a better understanding of the dynamics of TME and aid in identifying immunomodulatory targets for anti-cancer therapies. Therefore, the present study aims to analyze the distribution of CD4+ and CD8+ TILs in OSCC TME and their relationship to various clinicopathological parameters.

## Materials and methods

Study groups and design

This cross-sectional study was performed using archived formalin-fixed paraffin-embedded tissue samples obtained from 75 patients with OSCC who underwent surgery between 2018 and 2021 at Karpaga Vinayaga Institute of Dental Sciences, Chengalpet, Tamil Nadu, after obtaining institutional human ethics committee approval. The clinicopathological data of the patients including the age, site, gender, clinical stage, grade of the tumor, and the type of tumor invasion were obtained from the clinical records of the patients. Cases treated by radiotherapy or chemotherapy were excluded from the study. The sample size was calculated using G Power software (version 3.1.9.4) (Heinrich Heine Universitat, Dusseldorf, Germany). Using a mean difference between two independent groups with an error probability of 0.05, study power (1-b) of 80, and effect size of 0.72, a minimum sample of 62 was calculated, which was rounded to 75. The effect size was calculated using previous literature [5].

Tissue preparation and immunohistochemistry

For tissue preparation, 4 µm thick sections of OSCC tumor specimens were immune-stained for CD4 and CD8 antibodies independently. Briefly, sections were prepared in positively charged slides, deparaffinized, and rehydrated to water. Heat-induced antigen retrieval was performed with a pressure cooker in ethylenediaminetetraacetic acid buffer at pH 9.0. Further, endogenous peroxidase activity was blocked using a 3% hydrogen peroxide solution, and then incubated with pre-diluted anti-CD4 rabbit monoclonal antibody (CD4: EP204, Pathnsitu Biotechnologies, USA) and anti-CD8 mouse monoclonal antibody (CD8: C8/468, Pathnsitu Biotechnologies, USA), respectively. Later, the sections were incubated with PolyExcel HRP/DAB secondary antibody system (Pathnsitu Biotechnologies, USA) and, finally, counterstained with Mayer’s hematoxylin, dehydrated, cleared, and mounted with DPX. Human lymph nodes were used as positive controls, whereas the incubation in primary antibody was omitted in negative control slides.

Immunohistochemical evaluation

**Figure 1:**
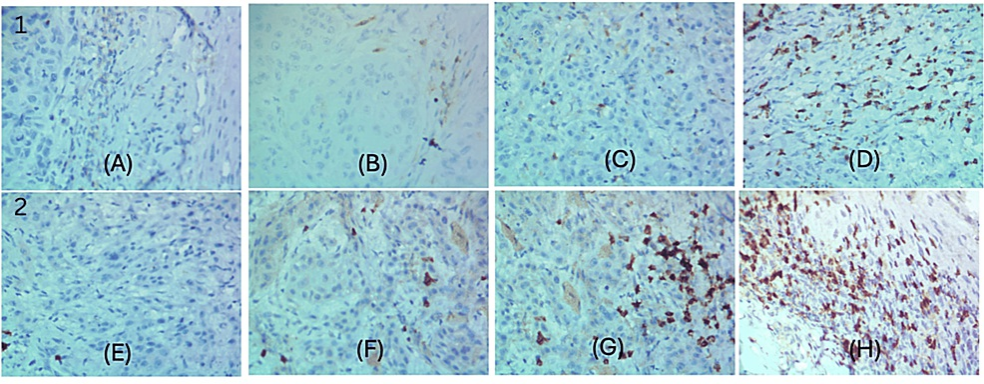
Scoring scale for the semi-quantitative analysis of immunohistochemical staining of CD4+ and CD8+ tumor-infiltrating lymphocytes. (1) CD4+ expression: (A) No staining, (B) 5%-35% of cells are stained, (C) 36%-75% of cells are stained, (D) >75% of cells are stained. (2) CD8+ expression: (E) No staining, (F) 5%-35% of cells are stained, (G) 36%-75% of cells are stained, (H) >75% of cells are stained.

Two trained and independent observers blinded to the clinical outcomes interpreted the slides. CD4+ and CD8+ cell expressions were evaluated semi-quantitatively by modifying the scoring system suggested by Wirsing et al. [6]. Five hotspots were selected at low power (×100) from the invasive front of the tumors. Every second visual field was selected at high power (×400) and the positively stained cells were counted and scored as follows: 0 if cells were immunonegative; 1 if 5%-35% of cells were stained; 2 if 36%-75% of cells were immunopositive; and 3, if >75% of cells were stained. The median score was calculated for each sample (Figure 1).

Statistical analysis

The data were compiled in Microsoft Excel (Microsoft Corp., Redmond, WA, USA) and subjected to statistical analysis using SPSS version 22 (IBM Corp., Armonk, NY, USA). Simple descriptive statistics were used for normal quantitative data, and frequencies were used for qualitative data. The bivariate relationship was displayed in cross-tabulations. The data distribution contained skewness and normality was not evident. Spearman’s correlation coefficient test was used to evaluate the association between CD4+ and CD8+ expression and clinicopathological parameters. Statistical significance was defined as p-values <0.05.

## Results

Patient characteristics

The study sample included 49 (65.3%) males and 26 (34.7%) females. The mean age was 50 years. The most common site of tumor occurrence was the tongue (n = 22, 29.3%), followed by the buccal mucosa (n = 20, 26.7%), lip (n = 9, 12%), retromolar trigone (n = 7, 9.3%), the floor of the mouth (n = 6, 8%), maxilla (n = 3, 4%), angle of the mouth (n = 2, 2.7%), palate (n = 2, 2.7%), hypopharynx (n = 2, 2.7%), gingiva (n = 1, 1.3%), and mandible (n = 2, 2.7%). In total, 13 (17.3%) patients had stage I cancer, 20 (26.7%) had stage II cancer, 27 (36%) had stage III cancer, and 15 (20%) had stage IV cancer (Table 1).

**Table 1:** Frequency and distribution of clinicopathological parameters.

Parameters	Frequency	Percent
Age (years)		
31–50	29	38.7
51–70	40	53.3
>70	6	8
Sex		
Male	49	65.3
Female	26	34.7
Tobacco smoking/Chewing		
Absent	38	50.7
Tobacco smoking present	32	42.7
Tobacco chewing present	5	6.7
Alcohol		
Absent	32	42.7
Fewer than 1 time weekly	15	20
More than 1 time weekly	28	37.3
Tumor site		
Maxilla	3	4
Buccal mucosa	20	26.7
Tongue	22	29.3
Mandible	1	1.3
Lip	9	12
Palate	2	2.7
Retromolar trigone	7	9.3
Gingiva	1	1.3
Floor of the mouth	6	8
Angle of the mouth	2	2.7
Hypopharynx	2	2.7
TNM stage		
Stage I	13	17.3
Stage II	20	26.7
Stage III	27	36
Stage IVA	15	20
Histopathological grade		
Well-differentiated squamous cell carcinoma	56	74.7
Moderately differentiated squamous cell carcinoma	17	22.7
Poorly differentiated squamous cell carcinoma	2	2.7
Perineural invasion		
Absent	65	86.7
Present	10	13.3
Lymphovascular invasion		
Absent	67	89.3
Present	8	10.7
Pattern of invasion		
Cohesive	32	42.7
Non-cohesive	34	45.3
Dispersive	9	12
Lymph node invasion		
Absent	63	84
Present	12	16

The tumors were histopathologically graded based on the World Health Organization grading system into well-differentiated (n = 56, 74.7%), moderately differentiated (n = 17, 22.7%), and poorly differentiated (n = 2, 2.7%).

Association between CD4+ and CD8+ cells in the tumor microenvironment and their association with various clinicopathologic parameters

When the CD4+ and CD8+ expression was compared, a significant positive correlation was noted (r = 0.655, p = 0.001) (Table 2).

**Table 2:** Association between CD4+ and CD8+ expression. **: significant positive correlation.

Correlation between parameters		r value	P-value
CD4+ expression	CD8+ expression	0.655	0.001**

When both expressions were compared with clinical parameters such as age, gender, type, tobacco habit, and site of the lesion, there was no statistically significant difference in the expression pattern. However, the expressions were negatively correlated with increasing stage of cancer (r = -2.37, p = 0.041 and r = -0.348, p = 0.002, respectively). When compared with the histopathological grading of the tumor, CD8+ expression had a positive correlation with increasing grade (r = 0.288, p = 0.012). Other histopathologic parameters such as perineural invasion, lymphovascular invasion, or pattern of invasion did not show any correlation for both CD4+ and CD8+ expressions (Table 3).

**Table 3:** Correlation between CD4+ and CD8+ expression and clinicopathological parameters. *: positive correlation; **: negative correlation.

		Age	Sex	Tobacco smoking/Chewing	Alcohol	Tumor site	TNM stage	Histopathological grade	Perineural invasion	Lymphovascular invasion	Pattern of invasion	Lymph node invasion
CD4	r	-0.142	0.066	-0.086	-0.145	0.026	-0.237*	0.129	-0.135	-0.003	0.071	-0.158
	p	0.225	0.572	0.463	0.213	0.827	0.041	0.272	0.249	0.978	0.544	0.175
CD8	r	-0.061	0.066	-0.047	-0.041	-0.2	-0.348**	0.288*	-0.052	0.009	0.04	-0.017
	p	0.605	0.573	0.687	0.73	0.085	0.002	0.012	0.656	0.94	0.736	0.886

## Discussion

The immune microenvironment primarily comprises TILs such as T lymphocytes, B lymphocytes, and natural killer cells [7]. Anti-tumor immunity is represented by varied subsets of TILs such as CD4+ and CD8+ T cells [2,3,8]. CD4+ T lymphocytes, also known as T helper cells, are versatile multifunctional cells that coordinate adaptive T-cell immunity. They recognize peptide antigens of Class II MHC II molecules. CD4+ T cells can either have an effector function and support differentiation into CD8+ T cells or differentiate into induced Tregs that mediate immunosuppression [2,9-13]. CD8+ T lymphocytes, also known as cytotoxic T cells, are potent anti-cancer immune effectors. They recognize peptide antigens in Class I MHC I molecules. CD8+ T cells inhibit cancer growth, and in the majority of cancers, CD8+ T cells are associated with favorable prognosis and disease-free survival [2,13-18].

The present study assessed the relationship between CD4+ and CD8+ TILs in OSCC, as well as their association with various clinicopathological features. A significant positive correlation was noted between the CD4+ and CD8+ expression levels. This is in accordance with a similar study by Lequerica-Fernández et al. [5], who reported a strong positive correlation between tumor CD4+ and CD8+ TILs in OSCC. This can be explained by the fact that CD4+ TILs play a key role in the upregulation of CD8+ TILs. They secrete interleukin-2 which stimulates the effector function, proliferation, and differentiation of CD8+ TILs. CD4+ cells are essential for maintaining and sustaining CD8+ TIL-mediated anti-tumor responses [9,19,20].

In this study, CD4+ and CD8+ expression was not significantly associated with clinical parameters such as age, gender, tobacco habit, or the tumor site. The results of the study were similar to those of a study by Wahbi and Manadili [8], where it was reported that CD4+ and CD8+ expression was not significantly associated with age, gender, and tobacco smoking. However, this is contrary to a study by Lequerica-Fernández et al. [5], in which there were higher CD4+ and CD8+ TILs in patients without tobacco smoking and alcohol consumption. This further suggests that tumorigenesis may be multifactorial and not limited to habits and tumor sites.

This study revealed that CD4+ and CD8+ expression was negatively correlated with the TNM stage. In a similar study by Mukherjee et al. [21], high counts of CD4+ cells at the invasive tumor front negatively correlated with tumor size and lymph node metastasis. Fang et al. [22] investigated the prognostic significance of TILs in OSCC and concluded that higher CD8+ expression was associated with the absence of regional lymph node metastasis. In contrast, in the study by Wahbi and Manadili [8], CD4+ and CD8+ infiltration did not show any significant association with the TNM stage. Similarly, Mohajertehran et al. [23] reported no significant association between CD4+ infiltration and the TNM stage. This difference in results could be due to the differences in the TIL scoring criteria, diverse tissue sampling, and variations in lymphocyte characterization.

In this study, CD8+ expression positively correlated with the histopathological grade of OSCC. This is consistent with studies by Santos et al. [24] and Mahmoud et al. [25], who reported increased CD8+ cells in higher grades of various cancers. CD8+ cells are potent components of adaptive immunity and exert a tumoricidal effect via an interferon-gamma-dependent mechanism [17,26,27]. The higher grade of tumors despite heavy CD8+ infiltration may be due to cancer immunoediting [25]. Cancer immunoediting is a dynamic process that shapes tumor immunogenicity. It consists of the following three phases: (i) elimination, (ii) equilibrium, and (iii) escape. In the elimination phase, innate and adaptive immune cells detect and eliminate cancer cells before they become clinically evident. During the equilibrium phase, the adaptive immune cells control tumor cell growth of uneliminated cancer cells. In the escape phase, the edited tumors with poor immunogenicity grow exponentially in an immunosuppressive TME and become clinically evident. Cancer immunoediting most likely occurs due to genetic and epigenetic changes in the tumor cell which confers it with the resistance to immune recognition and elimination [27].

There are some limitations of the current study. First, a larger sample size may show a significant association between CD4+ and CD8+ infiltration and the TNM stage. Second, varied lymphocyte characterization may significantly affect the association between CD4+ and CD8+ infiltration and clinicopathological parameters. Third, the characteristics of the cases concerning age, gender, habit usage, tumor site, lymphovascular invasion, pattern of invasion, TNM staging, and lymph node invasion were skewed.

## Conclusions

The study findings demonstrate that there was a significant positive correlation between CD4+ and CD8+ expression levels. CD4+ cells are essential for maintaining and sustaining CD8+ TIL-mediated anti-tumor responses. CD4+ and CD8+ expressions were negatively correlated with the TNM stage of OSCC. CD4+ and CD8+ TILs are key players in cell-mediated adaptive immunity and prevent tumor progression and metastasis. In comparison, CD8+ expression positively correlated with the histopathological grade of OSCC. CD8+ cells have a tumoricidal effect on cancer cells via an interferon-gamma-mediated mechanism. The higher grade of tumors despite heavy CD8+ infiltration may possibly be due to cancer immunoediting. Further research with a larger sample size will validate these results.
